# Interkingdom signaling between gastrointestinal hormones and the gut microbiome

**DOI:** 10.1080/19490976.2025.2456592

**Published:** 2025-01-24

**Authors:** Xinyu Zhao, Ye Qiu, Lanfan Liang, Xiangsheng Fu

**Affiliations:** Department of Gastroenterology, Clinical Medical College and the First Affiliated Hospital of Chengdu Medical College, Chengdu, Sichuan, China

**Keywords:** Gastrointestinal hormones, gut microbiota, interkingdom signaling, metabolic disease, dysbiosis

## Abstract

The interplay between the gut microbiota and gastrointestinal hormones plays a pivotal role in the health of the host and the development of diseases. As a vital component of the intestinal microecosystem, the gut microbiota influences the synthesis and release of many gastrointestinal hormones through mechanisms such as modulating the intestinal environment, producing metabolites, impacting mucosal barriers, generating immune and inflammatory responses, and releasing neurotransmitters. Conversely, gastrointestinal hormones exert feedback regulation on the gut microbiota by modulating the intestinal environment, nutrient absorption and utilization, and the bacterial biological behavior and composition. The distributions of the gut microbiota and gastrointestinal hormones are anatomically intertwined, and close interactions between the gut microbiota and gastrointestinal hormones are crucial for maintaining gastrointestinal homeostasis. Interventions leveraging the interplay between the gut microbiota and gastrointestinal hormones have been employed in the clinical management of metabolic diseases and inflammatory bowel diseases, such as bariatric surgery and fecal microbiota transplantation, offering promising targets for the treatment of dysbiosis-related diseases.

## 1. Introduction

Many recent studies have probed the close link between the gut microbiota and the health of the host, highlighting the ability of the microflora to shape host metabolic activity, immune activity, nervous system functionality, and other key factors.^1–4^ Gastrointestinal hormones are major regulators of metabolism and overall intestinal functionality and have increasingly been found to impact the gut microflora.^5–7^ Gastrointestinal hormones include glucagon-like peptide-1 (GLP-1),^8^ cholecystokinin (CCK),^9^ and peptide tyrosine tyrosine (PYY),^10^ and they are capable of regulating processes such as appetite,^10^ insulin sensitivity,^8^ and gastrointestinal motility to control energy balance and metabolism within the host.^11^ These hormones can also impact the overall makeup of the intestinal microbiota and its metabolic activity.^6,7^ As such, detailed analyses of how gastrointestinal hormones and the gut microbiome interact have the potential to inform new efforts to detect, prevent, and treat myriad forms of gastrointestinal disease.

## 2. Function and secretion of gastrointestinal hormones

Enteroendocrine cells (EECs), including G, D, I, K and L cells, are key components of the intestinal and gastric mucosa that are capable of producing more than 20 different bioactive compounds and hormones, including PYY, GLP-1, CCK, and serotonin^12–14^ (Table 1). In addition to their effects on gastrointestinal mobility, absorption, and secretion, these cells can also influence other tissues and organs through bloodstream-mediated signaling, ultimately shaping diverse physiological processes.^15^

**Table 1.** Overview of gastrointestinal hormones.

Gut hormone	Location of secretion	Endocrine cells	Function	References
VIP	Ileum and colon	D -cells	relaxes digestive tract smooth muscle	^21^
Ghrelin	Stomach	X/A -cells	promotes motilin release and increases appetite	^22^
Leptin	Stomach	P-cells	Inhibits appetite	^23^
Gastrin	Stomach and duodenum	G-cells	Promotes digestive juice secretion and gastric emptying	^24^
bombesin	Stomach	P-cells	Stimulates the release of gastrointestinal hormones	^25^
somatostatin	Stomach and Pancreas	D-cells	Inhibits hormone secretion and gastrointestinal motility	^26^
Secretin	Duodenum and jejunum	S-cells	Promotes digestive juice secretion	^27^
GLP-1	small intestine	L-cells	Promotes the secretion of insulin, and inhibits appetite and gastric emptying	^8^
CCK	Proximal small intestine	I-cells	Promotes bile acid release, gastrointestinal motility and appetite	^28^
Motilin	Duodenum and jejunum	M-cells	Promotes gastrointestinal motility	^29^
Enteroglucagon	small intestine	L-cells	Promotes glycogen decomposition and gluconeogenesis.	^30^
PYY	Ileum and colon	L-cells	Inhibits gastric emptying and appetite.	^31^
5-HT	Whole gastrointestinal tract	E-cells	Promotes gastrointestinal motility and appetite.	^32^
Catecholamines	Whole gastrointestinal tract	Pheochromocyte and sympathetic neurons	Increases gastric acid secretion and gastrointestinal peristalsis.	^33^
Pancreatic polypeptide	Pancreas	PP- cells	Promotes digestive juice secretion	^34^
GDF-15	Whole gastrointestinal tract	macrophages, adipocytes	Inhibits appetite and food intake	^35^
Famsin	Intestine	Unknown	Promotes energy mobilization.	^36^
Cholesin	Intestine	Intestinal cells expressing NPC1L1	Reduces cholesterol synthesis	^37^

A majority of the EECs found in the gastrointestinal tract extend microvilli into the lumen such that they can directly sense available nutrients and secrete appropriate hormones in response.^12,16^ However, rare EEC subsets that lack these microvilli can also be found closer to the basement membrane where they are believed to exhibit greater sensitivity to paracrine signaling and mechanical stimulation, potentially playing roles distinct from those of other EEC types.^16^ Different EEC types vary in their distributions throughout the gastrointestinal tract, with the X/A, G, D, and EC cell types, for example, primarily being present within the stomach, whereas the G, D, I, K, L, and EEC cell populations are also found in the small intestine, and L cell and EEC populations are found in the large intestine.^17,18^

These distribution profiles enable EECs to perform particular physiological functions conducive to the appropriate maintenance of stable gut health.^11^ Furthermore, G protein-coupled receptors (GPCRs) expressed on EEC surfaces also closely regulate gastrointestinal hormone secretion, ensuring that this secretory activity is responsive to a range of pathological and physiological stimuli such that the overall function of the gastrointestinal tract can be preserved in a context-dependent manner.^19,20^

## 3. Gut microbiome composition and function

The endogenous microflora found within the human gastrointestinal tract consists of more than 1,000 microbial species including eukaryotic cells, bacteria, viruses, archaea, and other organisms.^38^ Over 90% of these microbes are members of the Firmicutes and Bacteroidetes bacterial families.^39^ The symbiotic mutual interdependence that exists between these microorganisms and the host is vital for maintaining host health and preventing disease.^3^

**Figure 1.**
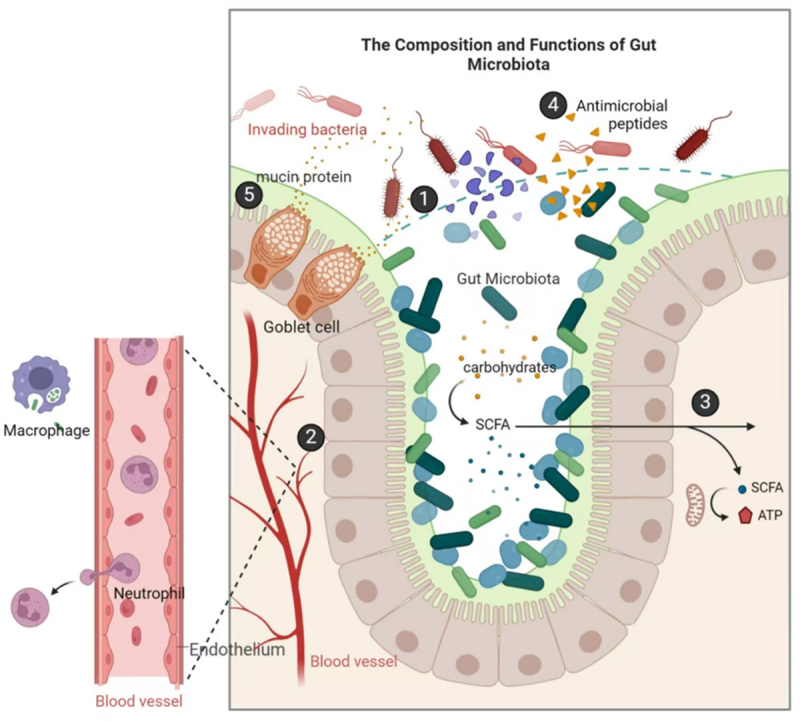
Gut microbiome functions. 1. Biological barrier formation. 2. Regulation of local angiogenesis and enhancement of immune function. 3. Production of energy sources. 4. Antimicrobial compound synthesis. 5. Stimulation of mucin secretion.

There are several key interactions that highlight the complex nature of the beneficial interactions between the commensal microbiome and the host (Figure 1). The ability of these symbiotic microbes to bind firmly to the intestinal mucosa produces a strong biological barrier that can protect against pathogen growth or the effects of toxins, thereby supporting intestinal stability.^40^ This tight adherence to intestinal epithelial cells by these microbes can also shape the proliferation and differentiation of these cells and local angiogenic activity, ultimately leading to enhanced intestinal immunity.^41^ Microbe- derived short-chain fatty acids (SCFAs) can also serve as valuable sources of energy for the cells of the intestinal epithelium,^42^ whereas secreted antimicrobial compounds can prevent harmful bacterial growth and preserve homeostatic balance within the gut microbiome.^43^ These commensal organisms can also stimulate the secretion of mucins from the gastrointestinal mucosa, ensuring appropriate intestinal lubrication while also reducing the risk of disease.^44^

## 4. Mechanisms of interkingdom signaling between microorganisms and the host

Interkingdom signaling between microorganisms and the host is mediated by metabolites derived from the gut microbiota and hormones from gastrointestinal cells. This encompasses the SCFAs pathway, the secondary bile acid pathway, LPS signaling and so on, establishing a subtle network within the microbe-host interaction through the regulation of hormone synthesis and release.

### 4.1. SCFAs: crucial mediators of the gut microbiota-host interaction and its regulatory mechanisms

SCFAs serve as primary mediators of the interactions between the host and the gut microbiome, with over 90% of SCFAs being produced by gut microbes as metabolites of difficult-to-digest carbohydrates.^45^ In addition to serving as a primary source of energy for cells of the intestines, SCFAs can help maintain immune activity, host metabolism, and overall gut homeostasis.^45–50^

Several factors shape the types and amounts of SCFAs products produced within the gastrointestinal tract. Bacterial species differ in their ability to utilize and degrade particular carbohydrates, giving rise to a diverse array of SCFAs.^51^ Intestinal transit time is another key consideration, as shorter transit times generally lead to the incomplete breakdown of carbohydrates, ultimately impacting the production of SCFAs.^52^ The presence of fiber in the diet of the host can also modulate bacterial carbohydrate utilization and SCFAs biogenesis in the gut.^53^

**Figure 2.**
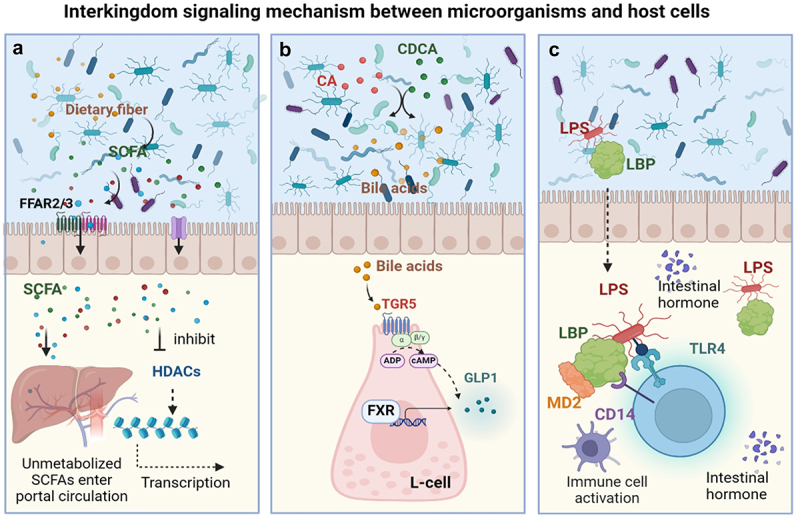
Mechanisms of interkingdom signaling between microbes and host cells. (a) SCFAs trigger gastrointestinal hormone release. (b) Secondary BAs control hormone release by serving as signaling molecules. (c) The cellular detection of microbe-derived structural components.

SCFAs are the primary mediators of host-microbiome interactions, and they exert their signaling effects in the human body through two main mechanisms. (1) Inhibition of histone deacetylases (HDACs). SCFAs can inhibit the activity of HDACs, thereby altering gene transcription and expression.^54,55^ This mechanism is crucial for regulating cell function, gene expression, and overall metabolic balance.^47,48^ (2) Stimulation of G protein coupling with free fatty acid receptors 2 and 3 (FFAR2/3). SCFAs activate the G protein signaling pathway by binding to FFAR2 and FFAR3 (Figure 2a). FFAR2 and FFAR3 are expressed in different regional patterns throughout the gastrointestinal tract, suggesting that SCFAs may produce different physiological effects in different segments of the intestine.^56,57^ Notably, FFAR2 has equal affinity for acetate, propionate, and butyrate, whereas FFAR3 has a much lower affinity for acetate compared to propionate and butyrate.^58^ These findings suggests that the interaction between SCFAs and FFAR2/3 is influenced by a combination of receptor type and the abundance of specific metabolites.

### 4.2. Secondary bile acids: signaling molecules regulating host physiology and metabolism

As cholesterol metabolites, bile acids (BAs) are produced in the liver and stored in the gallbladder prior to their secretion into the duodenum. Approximately 95% of BAs undergo hepatic resorption via the enterohepatic circulation, whereas the intestinal microbiome is responsible for the conversion of the remaining 5% into secondary Bas.^59^ This conversion process entails microbe-mediated primary BA dehydroxylation, dehydrogenation, and deconjugation,^60^ ultimately giving rise to more than 50 secondary BAs while also affecting the chemical properties of these molecules such that they can serve as signaling molecules capable of binding to cellular receptors and exerting a range of biological effects on host physiology.^60^

The ability of secondary BAs to modulate physiological and metabolic activity is mediated by the G protein-coupled receptor 5 (TGR5) and the farnesoid X receptor (FXR).^61^ FXR is widely expressed in the liver and intestines, where it responds to BA binding by activating transcriptional processes associated with the control of glucose, fat, and cholesterol metabolism.^62,63^ TGR5 is a membrane receptor that is highly responsive to BAs stimulation, with secondary BAs-induced TGR5 activation leading to the initiation of Gas-adenylate cyclase-cAMP signaling.^64,65^ Through these processes of TGR5 and FXR binding, BAs are able to maintain host cholesterol and glucose homeostasis as well as lipid metabolism, in addition to controlling overall energy metabolism and inflammation^65^ (Figure 2b).

Microorganisms modulate the composition of the BA pool by regulating the synthesis, metabolism, and reabsorption of diverse Bas.^66,67^ The varied receptor affinities and binding strengths of BAs allow the microbiota to control the metabolic activity of the host.^68^ For example, gut microbes can increase the production of some secondary BAs with greater TGR5 agonist activity, leading to greater energy expenditure and a reduction in the incidence of obesity.^69^ Through FXR-mediated signaling, secondary BAs can also control insulin sensitivity and glucose metabolism, thus affecting type 2 diabetes onset and progression.^70^ In atherosclerosis, secondary BAs can drive the processes of reverse cholesterol transport and excretion, leading to the deposition of lower levels of cholesterol within vessel walls and associated shifts in atherosclerotic plaque formation.^71^

### 4.3. LPS: the messenger of the microbial world

Microbial structural components including membrane-associated lipopolysaccharides (LPS) and flagella can function as signaling molecules that are recognized by host cells.^72^ Upon bacterial invasion, LPS is readily released from these pathogenic cells whereupon it binds to the LPS-binding protein (LBP), which subsequently transports LPS to the membrane protein CD14 on certain immune cell populations.^73^ CD14, in turn, transports LPS to a protein complex composed of Toll-like receptor 4 (TLR4) and myeloid differentiation protein 2 (MD2).^74^ The signal transduction mediated by this complex leads to the activation of a host immune response directed against LPS, with MD2 assisting TLR4 in the specific binding and recognition of LPS.^75^ After binding to LPS, a conformational change within the intracellular domain of TLR4 results in the transmission of a signal into the cell interior, ultimately engaging in a series of immune responses aimed at bacterial clearance^76^ (Figure 2c). However, when not appropriately controlled these responses may be deleterious, contributing to conditions such as chronic inflammation and metabolic disease.^74^

LPS stimulation can activate immune cell populations including T cells, B cells, natural killer cells, and macrophages, promoting the production of a range of cytokines and other factors that shape and amplify the overall immune response.^77^ LPS can trigger the activation of the innate immune complement pathway, further helping to protect against inflammation and infection.^78^ Additionally, LPS is capable of protecting the integrity of hematopoietic tissues and promoting leukocyte adhesion, which is vital for the maintenance of an appropriate hematopoietic system.^79^ In the clinic, elevated LPS levels are commonly observed in the context of diseases, and they are particularly closely linked to insulin resistance and obesity.^80,81^

## 5. Interactions between gut microbes and gastrointestinal hormones

The correlations that are evident between bacterial distributions along the gastrointestinal tract and hormone secretion are likely a result of the complex interplay between these microbes and the host, with these interactions being vital to the regulation of host immunity, metabolism, digestion, and nutrient uptake.^1–3^

Bacterial abundance gradually increases along the intestinal tract from the stomach into the large intestine. In the highly acidic stomach microenvironment, the predominant bacteria include gram-positive anaerobes such as *Helicobacter pylori, Lactobacillus*, and *Streptococcus* species.^82^ While the microbial distributions of the duodenal and jejunal microflora are similar to those of the stomach, the composition of the gut microflora shifts beginning in the ileum, where anaerobic bacteria including *Bacteroides, Bifidobacteria, Enterococcus*, and *Eubacteria* species tend to dominate.^83^

**Figure 3.**
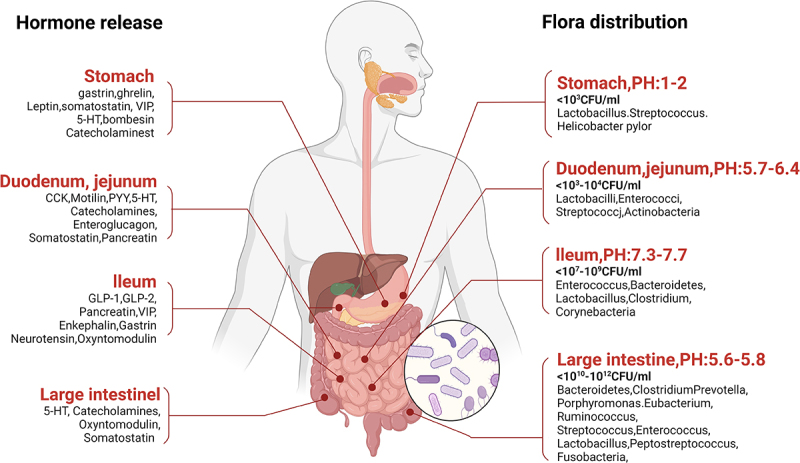
The distribution of gastrointestinal microbiota and the secretion of gastrointestinal hormones. The distribution of gastrointestinal microbiota varies with location, forming unique microbial ecologies; additionally, the secretion of gastrointestinal hormones finely regulates digestion and absorption functions. The distribution of microbiota and the secretion of hormones are anatomically intertwined, and their close interaction is crucial for maintaining gastrointestinal homeostasis.

Each segment of the digestive system participates in hormone secretion. The stomach secretes gastrin to promote gastric acid secretion and gastrointestinal motility,^24^ whereas somatostatin inhibits various digestive activities.^12^ In the duodenum, secretin and cholecystokinin aid in the secretion of pancreatic juice and bile,^27,28^ whereas motilin regulates gastrointestinal motility,^29^ with serotonin and substance P also playing a role in this region.^12^ The jejunum and ileum secrete peptide YY,^31^ and vasoactive intestinal peptide,^21^ which regulate the secretion of digestive enzymes, blood sugar and energy metabolism.^12^ Although the colon secretes relatively few hormones, its rich microbial community significantly influences systemic hormone levels and metabolic processes^20^(Figure 3).

A thorough understanding of both bacterial and hormonal dynamics is vital to gain a comprehensive understanding of the factors that shape gut physiology and pathology, with findings in this area having direct implications for human health. While the precise mechanisms through which microbial communities and host hormones interact are not fully understood, a large body of evidence indicates that these microbes and host hormone metabolism reciprocally influence one another through a bidirectional process^5,84–86^(Table 2).

**Table 2.** Interactions between gut microbiota and gastrointestinal hormones.

Interactions	Action	Mechanisms	References
Microbiota on hormones	Modulation of the intestinal environment	Intestinal oxygen level, pH, etc.	^87,88^
	Production of metabolites	SCFAs, gases, etc.	^89,91^
	Regulation of neurotransmitters	Excitatory or inhibitory neurotransmitters	^96,97^
	Integrity of the mucosal barrier	PSA and mucin expression, etc.	^101,104^
	Immune and inflammatory responses	Inflammatory mediators and T cell response.	^103,112^
Hormones on microbiota	Intestinal environment	Peristaltic activity, pH, etc.	^12,114^
	Absorption of nutrients	Carbohydrate or protein	^122,125^
	Bacterial metabolites	SCFAs, bile acids, etc.	^131,133^
	Biological behavior of bacteria	Bacterial virulence, proliferation, invasion, etc.	^147,148^

### 5.1. The gut microbiota influences gastrointestinal hormone synthesis and secretion

The regulatory effects of the gut microbiota on gastrointestinal hormones are intricate and involve multiple layers, such as the modulation of the gut microenvironment; bacterial metabolites; neurotransmitters; neuronal activities; the intestinal mucosal barrier; and immune and inflammatory responses. These comprehensive effects jointly ensure the balance of gastrointestinal hormones and the maintenance of host health.

#### 5.1.1. Modulation of the intestinal environment

Gut microbes can manipulate the characteristics of the gastrointestinal microenvironment through changes in oxygen levels,^87^ pH,^88^ and/or nutrient availability,^87^ thereby altering intestinal cell metabolic activity and indirectly altering hormones secretion.^88^

#### 5.1.2. Production of metabolites

Through their metabolic processing of a range of substrates, microbes can generate compounds including SCFAs,^89^ neurotransmitters,^90^ and gases,^91^ that are capable of indirectly or directly affecting intestinal cell function and hormone secretion. Acetate and butyrate, for instance, can stimulate glucagon-like peptide-1 (GLP-1) secretion from L cells,^92^ whereas the secretion of CCK can be induced by propionate.^93^

#### 5.1.3. Regulation of neurotransmitters release and neuronal excitability

Neurotransmitter release from gastrointestinal microbes and the modulation of intestinal neuronal excitability can enable the indirect modulation of gastrointestinal hormone secretion.^90,94^ *Bacillus subtilis, Escherichia coli, Lactobacillus plantarum*, Clostridium, Staphylococcus and *Enterococcus faecalis*, for example, can synthesize and release neurotransmitters such as serotonin,^95,96^ norepinephrine, catecholamines and dopamine,^97^ which can subsequently stimulate intestinal neuron activity to alter hormones productions. In contrast, *Lactobacillus rhamnosus* and *Bifidobacterium longum* can secrete ɣ-aminobutyric acid (GABA),^98^ an inhibitory neurotransmitter,^98^ and the sulfate-reducing bacteria *Methanobrevibacter smithii* and *Methanobrevibacter wolinii* can release metabolic byproducts that include methane and hydrogen sulfide,^99,100^ reducing secretory activity through the suppression of intestinal neuronal excitability.

#### 5.1.4. Through the regulation of the nervous system

By interacting with the enteric nervous system, intestinal microbes can impact gut neuronal activity and communication via the gut-brain axis, providing another avenue through which intestinal hormone production can be controlled.

#### 5.1.5. Effect on mucosal barrier integrity

The integrity of the gastrointestinal mucosa, as a barrier between the internal and external environments, is crucial for maintaining intestinal homeostasis. The intestinal commensal microbiota is an important component that maintains the integrity of the intestinal mucosa. Specific bacteria such as *Bacteroides fragilis* produce polysaccharide A (PSA) to promote immune tolerance and mucus stability.^101,102^ Probiotics such as *lactobacilli* and *Akkermansia* enhance mucin expression,^103,104^ fostering mucus layer repair and thickening, thereby creating a robust barrier and promoting hormone secretion.

When the intestinal microflora becomes unbalanced or provokes inflammation, it damage to the mucosal layer can occur, increasing its permeability.^105^ This, in turn, allows inflammatory mediators and bacterial factors to penetrate the submucosa, thereby stimulating intestinal gastrointestinal hormone secretion.^106^ A large subset of inflammatory bowel disease (IBD) patients, for example, present with an increase in mucosal permeability that may lead to excessive hormone and proinflammatory cytokine secretion from intestinal cells, resulting in disease exacerbation.^106^

#### 5.1.6. Regulation of immune and inflammatory responses

The gastrointestinal microbiota can alter intestinal immunity and inflammatory activity through immunomodulatory effects with the potential to alter the hormone sensitivity or responsiveness of intestinal cells.^107^ For instance, immune cell-derived inflammatory mediators may stimulate the secretion of gastrin,^108^ ghrelin,^109^ and other hormones from intestinal cells.^110^ There is also some evidence suggesting that these microbes can modulate gastrointestinal hormone release through the regulation of T cell responses within the intestine.^111–113^

### 5.2. Gastrointestinal hormones alter intestinal flora composition and activity

Gastrointestinal hormones can have reciprocal effects on the composition and function of the gut microbiota by finely regulating the intestinal environment, nutrient absorption, microbial community structure, metabolic activities, and bacterial biological behaviors.

#### 5.2.1. Regulation of the intestinal environment

Gastrointestinal hormones can alter the local pH within the intestines,^114^ peristaltic activity,^115^ and mucus secretory functions,^116,117^ all of which can affect gut microbe growth and metabolism. Gastrin and secretin can directly stimulate parietal cells in the stomach and exocrine glands in the pancreas to secrete gastric acid and pancreatic juice respectively, which contain acidic substances (such as hydrochloric acid) or alkaline substances (such as bicarbonate), thereby affecting the pH value of the intestinal tract.^11,12^ An acidic intestinal environment, for instance, is conducive to the growth of the probiotic species *Lactobacillus* and *Bifidobacterium*,^118^ whereas alkaline conditions can instead favor the growth of potentially pathogenic species including enterotoxigenic *Escherichia coli*^119^and *Vibrio cholerae*.^120^

#### 5.2.2. Modulation of nutrients absorption and utilization

Gastrointestinal hormones can further alter the composition of the intestinal microbiota by influencing the absorption and utilization of nutrients within the intestines. GLP-1 and CCK, for example, can enhance intestinal carbohydrate absorption,^9,121^ leading to altered *Bifidobacterium, Lactobacillus*, and *Bacteroides* growth owing to their dependence on unabsorbed carbohydrates as a source of energy.^122,123^ Gastrin and histamine stimulate the secretion of gastric acid and pepsin, promoting the digestion and absorption of proteins, which indirectly affects the growth and metabolism of intestinal bacteria.^124^ In the large intestine, bacteria utilize proteases and peptidases to degrade proteins and peptides into amino acids, providing nutrition sources for bacterial protein synthesis and energy production.^125,126^ Among these bacteria, *Bacteroides, Clostridium, Propionibacterium, Fusobacterium, Streptococcus*, and *Lactobacillus* play pivotal roles in the protein hydrolysis process in the large intestine.^127,128^

#### 5.2.3. Production of metabolites

Pancreatic polypeptide (PP) and secretin indirectly regulate the structure of the gut microbiota by stimulating the secretion of Bas.^129^ BAs, owing to their lipophilic properties, exhibit antibacterial characteristics, mainly by disrupting bacterial membranes and destabilizing macromolecules (RNA, DNA, proteins), thereby inhibiting the growth of some harmful bacteria.^130,131^ Models of biliary obstruction have shown that when Bas levels decrease, microbial proliferation and bacterial translocation increase,^132,133^ whereas supplementation with Bas can inhibit the excessive growth of intestinal bacteria.^132^ Additionally, bile acids exert an indirect antibacterial effect through the FXR-induced production of antimicrobial peptides and regulation of the host immune response.^134,135^ Different levels of bile acids significantly affect the composition of the gut microbiota,^136^ with low levels favoring the growth of gram-negative bacteria,^136,137^ and high levels favoring the growth of gram-positive *Firmicutes*.^136,137^

Furthermore, gastrin and motilin can stimulate gastrointestinal motility, accelerating the movement and mixing of intestinal contents, which enhances the contact between dietary fiber and the gut microbiota,^12^ leading to the production of more short-chain fatty acids (SCFAs).^138^ In turn, SCFAs further regulate the composition of the gut microbiota.^139^ Among them, propionic acid possesses antibacterial properties, inhibiting the growth of harmful bacteria such as bacillus spores;^140^ in contrast, butyrate promotes the growth of beneficial bacteria such as *Bifidobacterium* and *Lactobacillus*.^141^

#### 5.2.4. Regulating the biological behavior of bacteria

Chronic stress triggers the brain-gut axis, prompting the enteric nervous system to release gastrointestinal stress hormones, including norepinephrine, a neurotransmitter partially produced by the enteric nervous system itself and a pivotal mediator in stress responses.^142^ Norepinephrine exerts profound effects on the gut microbiota, fostering the growth of specific anaerobic bacteria, such as, *Bacteroides forsythus* and *E. coli*, enhancing their virulence traits.^143–146^

Our recent study revealed a positive correlation between norepinephrine and bacterial quorum sensing (QS) molecules in the progression of *Fusobacterium nucleatum (F. nucleatum)*-associated colorectal cancer. Under in vitro conditions, norepinephrine significantly upregulates QS-related genes in *F. nucleatum*, thereby potentiating its pathogenicity, and in vivo, norepinephrine accelerates the progression of colorectal tumors in *Apc*^Min+^ mice infected with *F. nucleatum* under stress conditions.^147^ Another recent study from our group revealed that norepinephrine can directly bind to the Quorum sensing regulator C of *F. nucleatum*, activating the expression of virulence genes such as FadA, FomA, and Fap2, promoting the invasiveness of *F. nucleatum* and aggravating colonic inflammation in IBD mice.^148^

### 5.3. Examples of the interaction between the intestinal flora and gastrointestinal hormones

#### 5.3.1. Serotonin

Serotonin (5-HT) is a product of tryptophan metabolism that is an important physiological regulator of activity within the human gastrointestinal tract.^149^ Enterochromaffin cells (ECs) are the primary source of 5-HT production in the gut.^150^ When released, 5-HT can regulate intestinal motility, modulate mucus secretion, promote vasodilation, and impact nutrient absorption to help maintain gastrointestinal homeostasis.^151^

**Figure 4.**
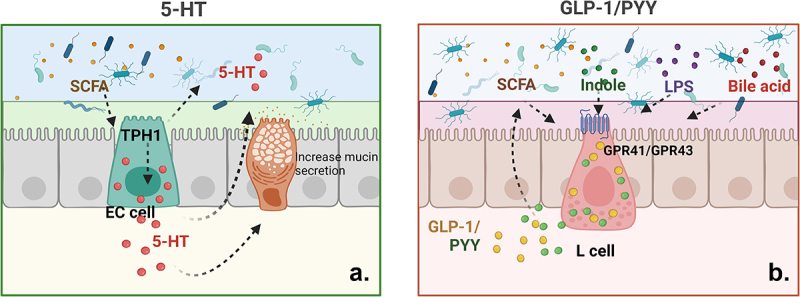
Interaction between major gastrointestinal hormones and the gut microflora.

The gut microbiome can have a strong effect on 5-HT secretion from EC cells through the key regulatory molecule tryptophan hydroxylase 1 (TPH1).^152^ SCFAs can also promote the upregulation of colonic TPH1 expression through their interactions with EC cells, leading to increased 5-HT synthesis^153^ (Figure 4a). Research has shown that microbial single-stranded RNA,^154^ the outer membrane protein Amuc_1100 of *Akkermansia muciniphila*,^155^ the CGA/ADRα2A signaling pathway of *Bifidobacterium*,^156^ and the compound SLPQ1 secreted by *Lactobacillus pentosus LPQ1*,^157^ effectively promote 5-HT production in the intestine.

(a) SCFAs derived from the intestinal microbiome promote colonic *TPH1* expression via interactions with EC cells, thereby driving the synthesis of 5-HT, which can influence gut microbiota composition through a range of mechanisms. (b) Intestinal microbe-derived SCFAs, BAs, and LPS can bind to the GPCRs GPR41 and GPR43 on L cells, stimulating GLP-1/PYY release and enhancing associated biological activity.

On the other hand, 5-HT affects the homeostasis of the intestinal flora through regulating intestinal epithelial secretion,^117^ motility and permeability,^158,159^ and promoting immune barrier function.^5,160^ For example, in vitro studies have shown that high concentrations of 5-HT inhibit the growth of symbiotic strains.^161^ A recent study reported that 5-HT tryptophan hydroxylase 2 knockout (TPH2 KO) mice exhibited disturbances in the colonic microbiota, specifically a decrease in *Lactococcus lactis* levels and an increase in *Proteobacteria levels*.^84^ Gastrointestinal dysbiosis may trigger abnormal serotonin production, leading to a range of diseases including inflammatory bowel disease,^162^ functional gastrointestinal disorders^163^ and gastroesophageal reflux disease.^163^

#### 5.3.2. GLP-1

Glucagon-like peptide-1 (GLP-1) is an intestinal hormone primarily produced by ileal and colonic L cells after meals, and it is highly responsive to glucose and other nutrients.^8^ Functionally, it can promote insulin secretion and inhibit the release of glucagon, thereby helping to maintain appropriate glucose homeostasis.^8^

Microbe-derived SCFAs can bind to GPR41 and GPR43 present on the surface of L cells, thereby inducing GLP-1 secretion and enhancing biological activity^164^ (Figure 4b). Other metabolites derived from the gut microflora may similarly provoke GLP-1 secretion. BAs, LPS, or indole-induced TGR5 activation, for instance, can stimulate GLP-1 production through binding to its cognate surface receptors.^165,166^ Recent studies have shown that some flora, such as *Akkermansia muciniphila* ^85^ and *Clostridium butyricum* ^167^ increase GLP-1 secretion, whereas other bacteria, such as *Clostridium perfringens*^168^ *Desulfovibrio* and *Bacteroides thetaiotaomicron* inhibit GLP-1 expression.^169^

Furthermore, significant changes in the gut microbiota composition were observed in type 2 diabetes patients treated with liraglutide, a GLP-1 receptor agonist.^86^ In obese mice, long-acting GLP-1 fusion peptide (MGLP_1) reduces the ratio of *Firmicutes* to *Bacteroidetes*, increases the abundance of beneficial genera (*Bifidobacterium, Lachnospiraceae*, and *Turicibacter*), and decreases the abundance of harmful genera (*Clostridium* and *Romboutsia*).^6^

An imbalance between the gut microbiota and GLP-1 activity subsequently influences metabolic homeostasis and immune function of the gut, contributing to the pathogenesis of type II diabetes,^86^ rheumatoid arthritis and chronic kidney disease.^170,171^

#### 5.3.3. PYY

PYY is an L cell-derived hormone similar to GLP-1 that is present primarily within the lower small intestine and colon.^10^ PYY binds to the Y2 receptors present on neuropeptide Y (NPY) neurons within the hypothalamic arcuate nucleus and agouti-related peptide neurons, thereby reducing appetite and food intake while promoting greater satiety.^10^

The SCFA butyrate can increase PYY gene expression in cultured human colonic cells.^172^ Additionally, secondary BAs have been shown to stimulate the secretion of PYY in many recent studies.^173–176^ PYY can in turn impact the gut microbiota. For example, a specific form of Paneth cell-derived PYY was recently identified as an antimicrobial peptide capable of preventing the transition of *Candida albicans* into its pathogenic form, thereby maintaining intestinal fungal homeostasis.^177^ Another study revealed that the abundance of *Alistipes*, *Bacteroides* and *Muribaculum* increased in PYY knockout mice.^7^

Imbalances between the gut microbiota and PYY dysfunction are involved in the progression of obesity,^10^ diabetes,^9^ intestinal infections,^177^ and inflammatory bowel diseases.^178^

#### 5.3.4. Leptin

Adipose tissue produces leptin, which plays key roles as a modulator of appetite, energy expenditure, and weight.^15^ Several complex interacting mechanisms shape the interplay between leptin and the gut microbiome. The administration of vancomycin or other antibiotics to rats has been shown to markedly decrease circulating levels of leptin, suggesting that the microbiome strongly impacts leptin synthesis and/or release.^179,180^ Positive correlations have been observed between leptin concentrations and the abundances of some bacterial genera including *Bacteroides, Lactobacillus, Bifidobacterium*, and *Lactococcus*,^181^ whereas negative correlations have been documented between leptin concentrations and the abundances of *Proteus, Clostridium, Bacteroides*, and *Bacillus*.^182,183^ The intestinal microbiota and leptin work synergistically to regulate energy balance and body weight, and their imbalance is closely related to metabolic diseases such as obesity, diabetes and metabolic-related fatty liver disease.^184^

## 6. Clinical practice

The microbiota, immunity and motility of the gut are closely related to certain intestinal disorders, such as IBD and IBS.^162,163,178^ In recent years, interventions of such, interactions through bypass surgery or microbiota transplantation have offered new approaches for the management of such diseases.

### 6.1. Gastric bypass surgery

Roux-en-Y gastric bypass (RYGB) has been a popular weight-loss surgery in recent years. In this surgery, doctors alter the normal path of food through the digestive tract to reduce food intake and absorption.^185^ Significant correlations have been detected between this form of surgery and changes in microbiome composition, hormone production, and metabolic activity.^186,187^ Postoperative increases in the relative abundance of phyla including *Enterobacteriaceae* (e.g., *E. coli*) and *Verrucomicrobia* (e.g., *Akkermansia*),have been described, with these changes being linked primarily to anatomical changes following surgery, indirectly leading to changes in the intestinal microbiome.^186^ Following RYGB surgery, the levels of hormones including insulin, GLP-1, and glucose-dependent insulinotropic peptide are significantly increased.^187^ These hormones can regulate appetite, energy metabolism, and blood sugar levels, contributing to weight loss and metabolic improvements.^187^

### 6.2. Fecal microbiota transplantation (FMT)

The novel FMT approach involves the transfer of fecal microbiome components from healthy subjects into the intestines of patients. The potential of this therapeutic modality to alter gastrointestinal peptide secretion and metabolic activity has been the focus of an increasing number of studies in recent years.^188,189^

Dysbiosis of the gut microbiota and the abnormal secretion of 5-HT are frequently observed in IBD patients, contributing to intestinal dysfunction (such as impaired motility and secretion) and visceral hypersensitivity symptoms.^190^ Given the intricate interplay between the gut microbiota and the 5-HT signaling system, FMT was recently utilized to restore the gut microbiota balance and modulate 5-HT levels, and represents an innovative and effective therapeutic approach for the treatment of IBD.^191^ Additionally, short bowel syndrome (SBS) is a condition in which extensive intestinal resection results in severe malabsorption, higher levels of *Lactobacilli* together with fecal lactate accumulation, and decreases in the abundance of certain anaerobes including *Clostridium* species.^192^ The transplantation of feces from short bowel syndrome patients into germ-free animals was shown to increase circulating gastrin and GLP-1 levels,^193^ suggesting that reshaping of the intestinal microbiome by FMT may lead to improved peptide secretion and metabolic activity in SBS patients.

## 7. Summary

The dynamic interplay between host-derived hormones and the gut microbiome remains an active field of research. Although the specific signaling pathways that characterize these interactions remain incompletely understood, a wealth of evidence highlights the vital role that commensal microbes play in shaping hormone metabolism, with these hormones having a reciprocal effect on the microbiota itself. These bidirectional effects ultimately establish a system that can preserve homeostatic balance through the control of physiological processes including immune activity, metabolic functionality, and appetite control. The management of such interactions provides a new avenue for maintaining host health and treating dysbiosis related diseases such as obesity, diabetes, and inflammatory bowel disease, thus implying new promising targets for treatment.
